# Accuracy of electronic apex locators to detect root canal perforations with inserted metallic posts: an ex vivo study

**DOI:** 10.1186/s13005-014-0057-2

**Published:** 2014-12-23

**Authors:** Benjamín Briseño Marroquín, Claudia Cortazar Fernández, Irene Schmidtmann, Brita Willershausen, Fernando Goldberg

**Affiliations:** Department of Operative Dentistry, Johannes Gutenberg University Medical Center, Mainz, Germany; Department of Endodontics, School of Dentistry, USAL-AOA, Buenos Aires, Argentina; Universidad Autónoma de Nuevo León, Monterrey, Nuevo León Mexico; Institute of Medical Biometrics, Epidemiology and Informatics, Johannes Gutenberg University Medical Center, Mainz, Germany; Poliklinik für Zahnerhaltung, Langenbeckstr. 1, Mainz, 55131 Germany

**Keywords:** Root perforation, Metal post, Diagnosis, Apex locators

## Abstract

**Introduction:**

The detection of possible root canal perforations caused during a metal post placement is frequently difficult to diagnose. The aim of this study was to investigate the accuracy of apex locators to diagnose such perforations.

**Materials and methods:**

Thirty mesiolingual root canals of extracted mandibular molars were instrumented (30/.04) and a post space was prepared. A root canal perforation was intentionally made at the bi-furcation (n = 15). Metal posts were cemented in 15 perforated and 15 non-perforated root canals. The teeth roots were embedded in an agar-agar solution. The resulting measurements (“short” or “beyond” the apex) disclosed if a perforation could be identified with five different apex locators (ProPex II, Elements Apex Locator, Apex NRG, Raypex 5 and Raypex 6). The sensitivity and specificity (95% interval confidence) were calculated.

**Results:**

All devices excluded the absence of perforations (100% with 95% confidence interval [78%; 100%] specificity). The Apex NRG and Raypex 6 detected all perforations (100% with 95% confidence interval [78%; 100%] sensitivity). The ProPex II, Elements Apex Locator, and Raypex 5 detected 14 out of 15 perforations (93% with 95% confidence interval [68%; 100%] sensitivity).

**Conclusions:**

All devices determined root canal perforations, due to metallic posts, within clinical acceptable ranges.

## Introduction

Despite technological advancements in endodontic techniques, endodontic mishaps such as root perforations during access preparation, root canal instrumentation, or preparation for post space are not unusual [1,2]. Root perforations compromise the success of endodontic therapy and have been regarded as, and probably are still, one of the most unpleasant accidents to deal with during re-treatment [3,4]. Occasionally, a clinician will be challenged with the fact that a radiological diagnose of patient with non-acute clinical symptomatology shows that the tooth has been previously endodontically treated, a post has been placed and the crown restored without evidence of a root perforation caused during the post insertion. A root perforation is defined as an artificial opening in the tooth crown or root area creating a communication between the root canal system with the periodontal tissues or oral cavity [5]. Iatrogenic root perforations are frequently caused by inappropriate post space preparation and have been classified as one of the most common types of root perforations [6] occurring approximately between 2 to 12% of the endodontically treated teeth [2,7]. The time elapsed between perforation and treatment [3,8,9], perforation size and location [10,11] play an important role when treating the affected site [3]. The treatment possibilities [4,5,11] as well as the post-treatment outcome of a root perforation [12] are decisive for their prognosis. Strömberg et al. [4] examined teeth that had been treated with different treatment modalities, such as a root-end resection, root amputation or hemisection and suggested a root perforation classification depending on the perforation location. In practice however, the indications for surgical correction of root perforations are being reduced by the improved non-surgical management of perforations with mineral trioxide aggregate type cements [13-15] and by the use of implants.

Root perforations, especially in the buccolingual aspect of the root are difficult to diagnose radiographically [16,17]. Since Sunada [18] introduced an electronic device to locate the apical foramen various reports [16,17,19] advocate the use of an apex locator as a tool to detect root perforations. Different authors [19-21] have suggested the employment of an apex locator to detect a root perforation when a metal post is placed. If effective, this precaution would allow the clinician to plan the endodontic re-treatment; thus, tooth disassembling prior to being astounded by the circumstances and having to immediately implement adequate treatment precautions. Thus, the aim of this study was to investigate with an ex-vivo model the accuracy of different electronic apex locators when diagnosing root perforations due to placement of metal posts.

## Materials and methods

**Figure 1:**
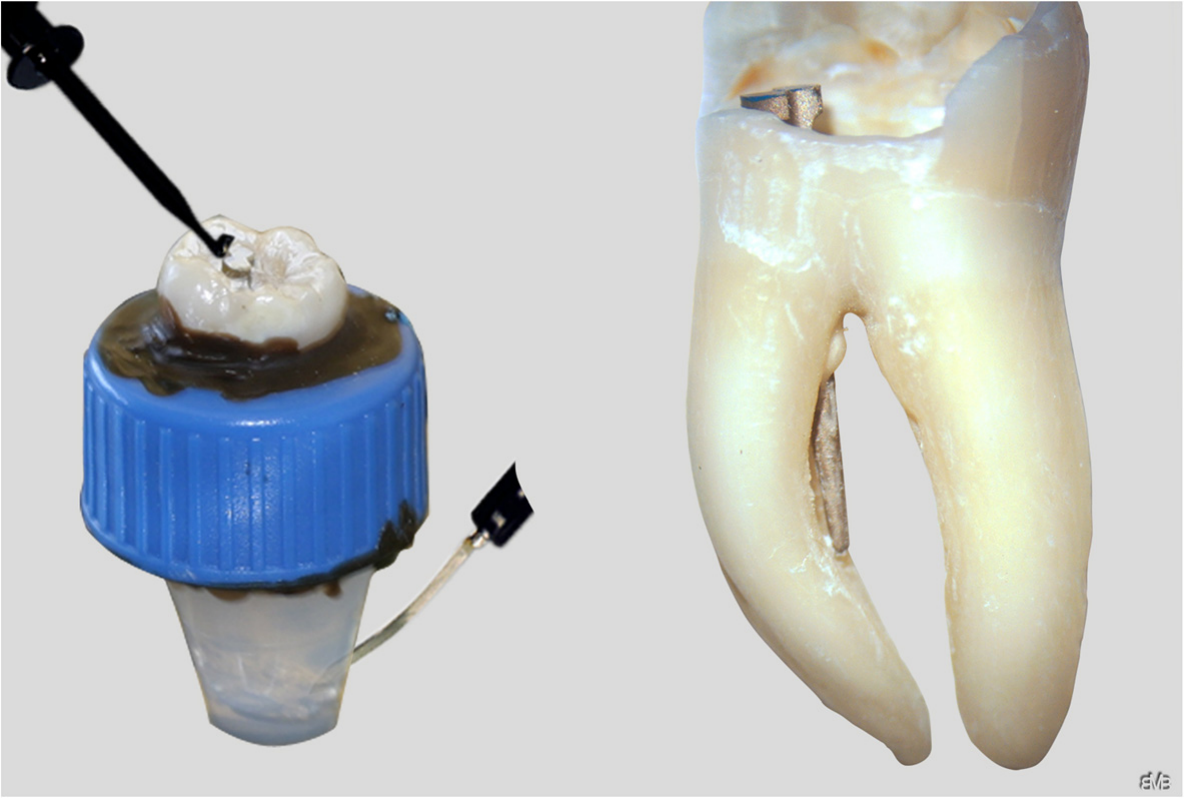
**Fixation device showing the root tooth placed in a plastic tube in an agar-agar solution and with both apex locator electrodes (left) and a maxillary molar with a perforated root canal through a metal post (right).**

An access cavity was prepared on 30 freshly extracted human first and second mandibular molars. Teeth with wide open apices or incomplete root development, root fracture or previous root canal treatments were excluded. Only mesiolingual root canals with an independent entrance at the pulp chamber floor and corresponding foramen were prepared to an instrument size 30/.04 (ProFile; Denstply Maillefer/Ballaigues, Switzerland). The actual working length of the mesiolingual root canals was determined when the tip of a K-type file size 10 (VDW/Munich, Germany) could microscopically (20x; Leica MZ6/Leica Microsystems, Wetzlar, Germany) be observed as it reached the physiological foramen [22]. The straight segment of the mesiolingual root canals was then prepared to receive a metallic post (Cytco-K/L50A; Maillefer Denstply/Ballaigues, Switzerland) with a Cytco-K drill (C 170 K 0 L50; Maillefer Denstply/Ballaigues, Switzerland). The teeth were then divided at random into two groups of 15 each. In one group a metallic post was conventionally cemented (Ketac Cem; 3 M Espe/Seefeld, Germany). In the other group a perforation in the bi-furcation area at the border between the coronal and middle thirds was intentionally made with a LN Bur #6 (ØISO 012; Maillefer Denstply/Ballaigues, Switzerland). The metallic posts were cemented with Ketac Cem (3 M ESPE/Seefeld, Germany) in the perforated root canals in such a way that the post tip segment was inserted beyond the perforation borders, thus allowing a contact with the periradicular tissue simulating media (Figure 1).

The teeth were fixed with wax in plastic tubes leaving the complete root length inserted in an solidified (20 min) agar-agar solution, which had a constant pH of 7.3 and simulated the periradicular tissue [19,23]. A lateral orifice in the plastic tubes allowed the placement of the negative rod of the electronic apex locators (Figure 1). The electronic measurements to diagnose the perforations were made by establishing a contact between the metal post and the EAL electrode by one operator in a blind manner (n = 30). It was expected that the measurements of the mesiolingual root canals with the metal posts indicated a “short measurement” when no perforation was present (n = 15) and a “beyond the apex” measurement when a perforation was existent (n = 15). The measurements were made with the Elements Apex Locator (SybronEndo/Orange CA, USA), ProPex II (Denstply Maillefer/Ballaigues, Switzerland), Apex NRG (Medic Energy, Tel Aviv, Israel) and Raypex 5 and Raypex 6 (VDW, Munich, Germany). The readings “short” or “beyond” the apex were made according to the respective display of the different EAL’s. All perforation diagnose measurements were made with each EAL at once. The sensitivity and specificity with respect to the gold standard and exact 95% confidence intervals for sensitivity and specificity were determined. Kappa statistics were computed to assess inter-device agreement when evaluating the presence or absence of root perforations between the different devices (SAS 9.3/2002–2010 SAS Institute Inc., Cary, NC, USA [25] and Multirater Kappa/SAS-Macro (Chen et al. 2005).

## Results

In all 15 mesiolingual root canals in which the metal post was within the root canal limits, all electronic devices showed a “short measurement”; thus, accurately excluding perforations when they were not present (100% specificity; 95% confidence interval [78%; 100%]). In the other group in which the post was placed beyond the perforation the Apex-NRG and Raypex 5 were able to detect all perforations (100% sensitivity; 95% confidence interval [78%; 100%]). The ProPex II, Elements Apex Locator and Raypex 6 showed 14 measurements “beyond the apex” and one “short measurement”; thus diagnosing 14 of 15 perforations and resulting in a 93% sensitivity (95% confidence interval [68%; 100%]) (Table 1). The calculated inter-device (Table 2) agreements showed to be high.

**Table 1:** **All methods excluded perforations when they were not present (100% specificity; 95% confidence interval [78%; 100%])**

**Device**	**Sensitivity with 95% confidence interval**	**Specificity with 95% confidence interval**
ProPex II	93% [68%;100%]	100% [78%; 100%]
Elements Apex Locator	93% [68%;100%]	100% [78%; 100%]
Apex NRG	100% [78%; 100%]	100% [78%; 100%]
RayPex 5	100% [78%; 100%]	100% [78%; 100%]
RayPex 6	93% [68%;100%]	100% [78%; 100%]

The Apex NRG and RayPex 5 also detected all perforations (100% sensitivity; 95% confidence interval [78%; 100%]). The ProPex II, Elements Apex Locator, and RayPex 6 detected 14 of 15 perforations (93% sensitivity; 95% confidence interval [68%;100%]).

**Table 2:** **Results of the inter-device agreement which proved to be high**

**Device 1**	**Device 2**	***κ*** ** with 95% confidence interval where possible**
Pro Pex II	Elements Apex Locator	0.86 [0.69; 1.00]
Pro Pex II	Apex NRG	0.93 [0.81; 1.00]
Pro Pex II	RayPex 5	0.93 [0.81; 1.00]
Pro Pex II	RayPex 6	0.86 [0.69; 1.00]
Elements Apex Locator	Apex NRG	0.93 [0.81; 1.00]
Elements Apex Locator	RayPex 5	0.93 [0.81; 1.00]
Elements Apex Locator	RayPex 6	0.86 [0.69; 1.00]
Apex NRG	RayPex 5	1.00
Apex NRG	RayPex 6	0.93 [0.81; 1.00]
RayPex 5	Raypex 6	0.93 [0.81; 1.00]

## Discussion

Root canal perforations are most of the time procedural errors and represent a serious treatment complication [3,4] that compromises the health of the periradicular tissues and adversely influences the retention of the tooth [3,26,27]. The detection of root perforations by means of an electronic apex locator has been in vitro [16,17] and in vivo [19] investigated. Even though the devices investigated by these researchers are from older generations, they are all in agreement, that root perforations can be confidentially diagnosed by means of an apex locator. Mesial root canals of mandibular molars were chosen for sake of research parameters standardization and since often the entrance morphology of distal root canal is quite oval; thus, making it difficult to accurately prepare the space for a prefabricated metal post. A limitation of this in vitro investigation method could be that it would be burdensome to extrapolate the results to different clinical conditions with the one investigated. However, the use an in vitro research method allows the possibility to investigate a relative high number of samples under the same parameters and reproducible conditions; thus, enabling a reliable statistical analysis. The employment of an agar-agar solution as embedding media was chosen since the methodology used in this investigation is an established and frequently employed one [23,28-31]. Different authors report that the use of alginate as embedding media showed a higher accuracy tendency [32] or a statistical significant higher accuracy [33] when compared with other embedding media such as agar-agar. However, in this last report the accuracy was not only embedding media but also apex locator dependent. Thus, the probability that using a different embedding media, such as alginate, could lead to different results is given and should be investigated.

In recent years non-surgical treatment [34,35] of root perforations has been facilitated by the use of magnification and illumination. Yet, the clinical and radiological diagnosis of root perforations is inherently complex [16,17]. Unfortunately, there is a lack of evidence-based research concerning the diagnosis of root perforations, in which a metal post has been placed, upon which treatment decisions can be based. Chong and Pitt Ford [20] suggested the use of an electronic apex locator as an aide to determine perforations in such clinical cases. It is possible that this method is widely used in clinical practice, yet, to the best of our knowledge, there are no scientific reports concerning such reliability. Different authors [16,17,19] have suggested the possibility to diagnose root perforations with apex locators. However, due to the different clinical conditions and the intrinsic limitations of clinical case reports, they can only be compared to a certain extent with the results of this investigation. Although the sensitivities obtained in this investigation were high, some EALs they did not reach 100%. The false measurements with the different apex locators were made in different teeth. A research methodological error can be excluded since the Raypex 6 and Apex NRG reached 100% sensitivity. Thus, it becomes a difficult task to point out a logical explanation for this situation. However, the sensitivity and specificity shown in the results suggest that any of the devices investigated are clinically reliable to diagnose these type of perforations.

A self-evident limitation of this method is that apex locators will not be able to detect a perforation when a fiber glass or a similar post has been inserted. Furthermore, the operator should be aware of the possibility that the luting cement could completely isolate the post from the periradicular tissues; thus, such clinical situation could compromise an accurate perforation diagnose. Although the root perforations in this investigation had a specific location, we are of the opinion that the detection of root perforations in which a metal post has been placed, either in the apical, middle or coronal root third could be as well diagnosed by means of apex locators. The method is simple; nonetheless, the removal of restoration materials surrounding the coronal part of the metal post, which could act as an isolating material, should be meticulously undertaken in order to be able to establish a stable contact between the apex locator electrode and metal post. The early detection during endodontic re-treatment of a root canal perforation caused by metal post would improve the clinical decision-making for both operator and patient.

## Conclusions

Under the conditions and with the electronic devices employed in this study we were able to determine a non-existent root canal perforation in all cases (100% specificity; −/−). The ProPex II, Elements Apex Locator and Raypex 6 had a 93% and the Apex NRG and Raypex 5 a 100% sensitivity (+/+). The high specificity and sensitivity and inter-device agreements obtained when diagnosing root perforations with a metal post placed in this investigation allow to recommend the use of an apex locator when diagnosing root perforations caused during the placement of a metal post.
